# Leber hereditary optic neuropathy following head trauma and ocular trauma on contralateral eye: a case report

**DOI:** 10.1007/s10633-020-09801-z

**Published:** 2020-10-17

**Authors:** Hoon Dong Kim

**Affiliations:** Department of Ophthalmology, College of Medicine, Soonchunhyang University, 30, Soonchunhyang 6-gil, Dongnam-gu, Cheonan-si, Chungcheongnam-do 31151 South Korea

**Keywords:** Leber hereditary optic neuropathy, Optic nerve, Trauma, Visual evoked potentials

## Abstract

**Purpose:**

To present a case of activation of Leber hereditary optic neuropathy (LHON) following head and ocular trauma of the fellow eye in the patient with no remarkable symptoms and normal visual acuity prior to trauma.

**Case summary:**

A 31-year-old healthy man was referred to our hospital after a traffic accident. He had blowout fractures of medial and inferior orbital wall of the left eye, subcutaneous hematoma of the left forehead, and bony fragment that compressed the left optic nerve. Initially, best-corrected visual acuity (BCVA) was 20/20 in the right and 20/1000 in the left eyes. Relative afferent pupillary defect of the left eye was apparent, and fundus examination revealed choroidal rupture circumferentially crossing the macular area. Nine months later, the patient complained with gradual vision loss in the right eye, which was the contralateral eye of the ocular trauma. BCVA was 20/200, and perimetry revealed cecocentral scotoma in the right eye. BCVA in both eyes reduced to 20/2000 1 year post-trauma. Visual evoked potentials revealed markedly decreased in amplitudes and elongated latencies for both eyes. Mitochondrial DNA analysis revealed a G11778A mutation; therefore, a diagnosis of activation of LHON followed by trauma was made for the previously unaffected carrier.

**Conclusions:**

This is a case in which activation of LHON occurred in a healthy carrier following head and ocular trauma of the fellow eye. This observation suggests the possibility that LHON activation in healthy carriers may occur in patients who experience head or ocular trauma even in the fellow eye.

## Introduction

Leber hereditary optic neuropathy (LHON) is a mitochondrially inherited disorder affecting retinal ganglion cells (RGCs), which results in the degeneration of cells and their axons. It eventually induces acute or subacute bilateral central visual loss in patients, and predominantly affects young men [1–3]. Several mutations in LHON patients have been reported, and a G11778A mutation is the primary mutation in patients worldwide and identified in 90% of the Asian population [3, 4].

Visual loss in LHON patients associated with head or ocular trauma has been reported only rarely, and varying time intervals from trauma to visual loss have been observed [1, 2]. Unaffected LHON healthy carrier can become symptomatic as a result of activating triggers including smoking, alcohol abuse, and antibiotics such as macrolide, aminoglycoside, ethambutol, isoniazid, and linezolid [1, 2]. Generally, brain magnetic resonance imaging (MRI) in LHON patients has been described as normal, but chiasmal enlargement of the optic nerve without contrast enhancement has been identified [1, 2]. Most previous reports have been described activation of LHON in the injured eye after direct ocular or head trauma.

In this report, a case of activation of LHON following head and fellow eye trauma in a patient with no remarkable symptoms and normal visual acuity prior to the trauma is presented with literature review. The informed consent was obtained from the patient.

## Case report

**Fig. 1:**
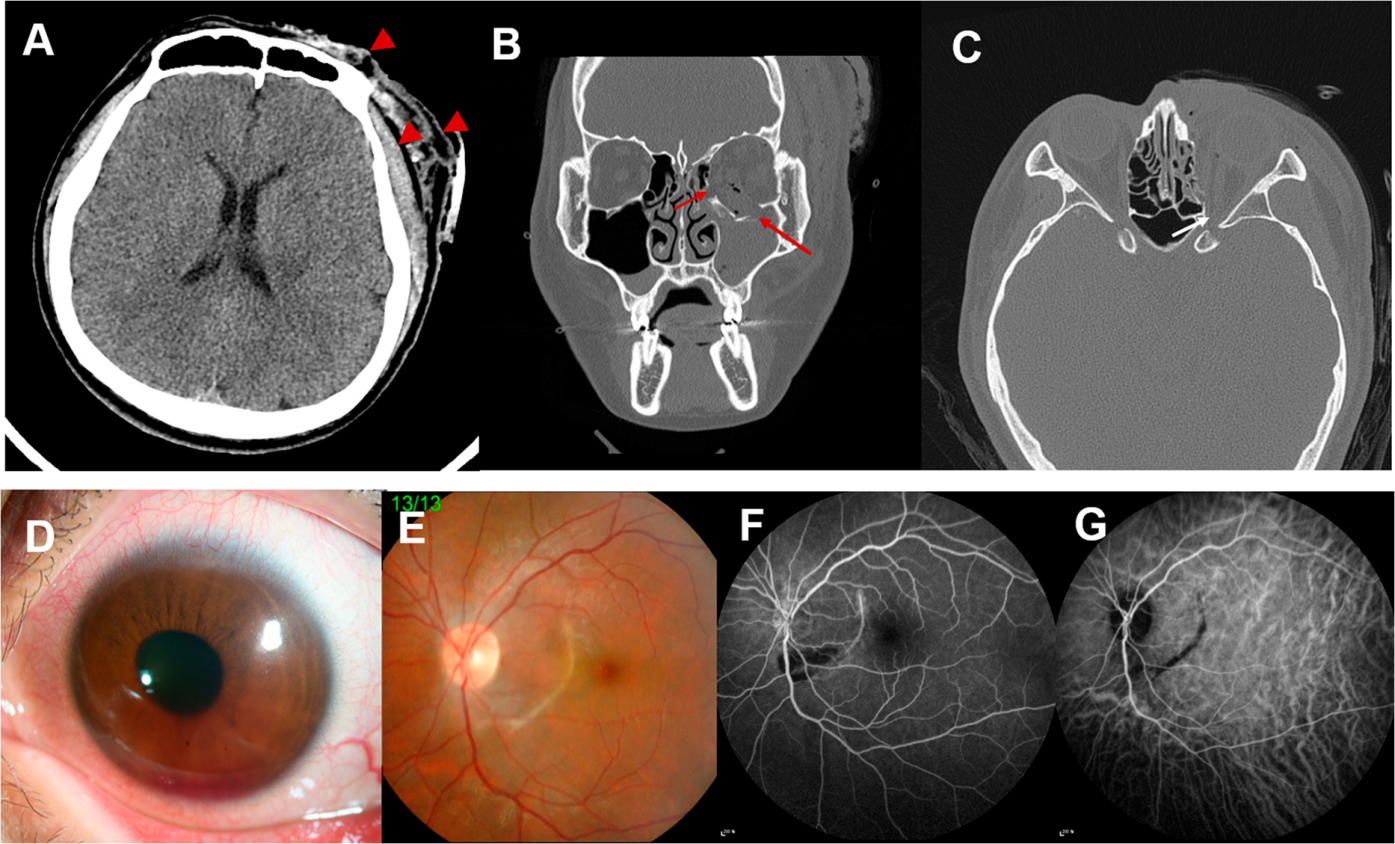
Initial brain and facial bone computed tomography (CT) images and ocular findings of the patient after traffic accident. Severe swelling and a subcutaneous hematoma are shown on the left forehead, the temporal area of the head, and the left periorbital area (**a**, red arrowheads). However, no evidence of skull fracture or intracranial hemorrhage is observed. The skull and brain parenchyma of the right side was spared (**a**). A blowout fracture of the medial and inferior orbital walls of the left eye was identified on axial and frontal CT scans (**b**, red arrows). Herniation of orbital tissue with hemorrhage is shown in the left maxillary sinus, and retrobulbar hemorrhage is also observed in the left orbital space. In addition, a bony fragment which compressed the optic nerve in the left eye is shown (**c**, white arrow). No signs of blowout fracture are observable in the right eye. Traumatic hyphema was found in the anterior chamber (**d**). Choroidal rupture (**e**), and hyperfluorescence in fundus fluorescein angiography (**f**) and hypofluorescence in indocyanine green angiography (**g**) on the corresponding area of choroidal rupture are shown

A 31-year-old healthy man was referred to the hospital after he was involved in a traffic accident. He had no documented history of systemic disease including hypertension, diabetes, or other genetic disorders. Also, he had no specific history about ophthalmologic conditions. A sagittal computed tomography (CT) scan of the brain revealed severe swelling and subcutaneous hematoma on the left forehead (Fig. 1a), but no evidence of skull fracture or intracranial hemorrhage was observed. He experienced blowout fractures of the medial and inferior orbital walls of the left eye (Fig. 1b), and a convulsive, full-thickness laceration on the left upper eyelid. Herniation of orbital tissue with hemorrhage was also identified in the left maxillary sinus, and a retrobulbar hemorrhage was revealed in the left orbital space. Additionally, bony fragment that compressed the left optic nerve was found in the left eye (Fig. 1c). On the other hand, no external wound in the right periocular area was observed, and no abnormal findings by as a result of trauma were observed in the periorbital soft tissue of his right eye via CT scan. Brain CT imaging revealed no abnormal findings such as optic nerve compression in the right eye, either.

**Fig. 2:**
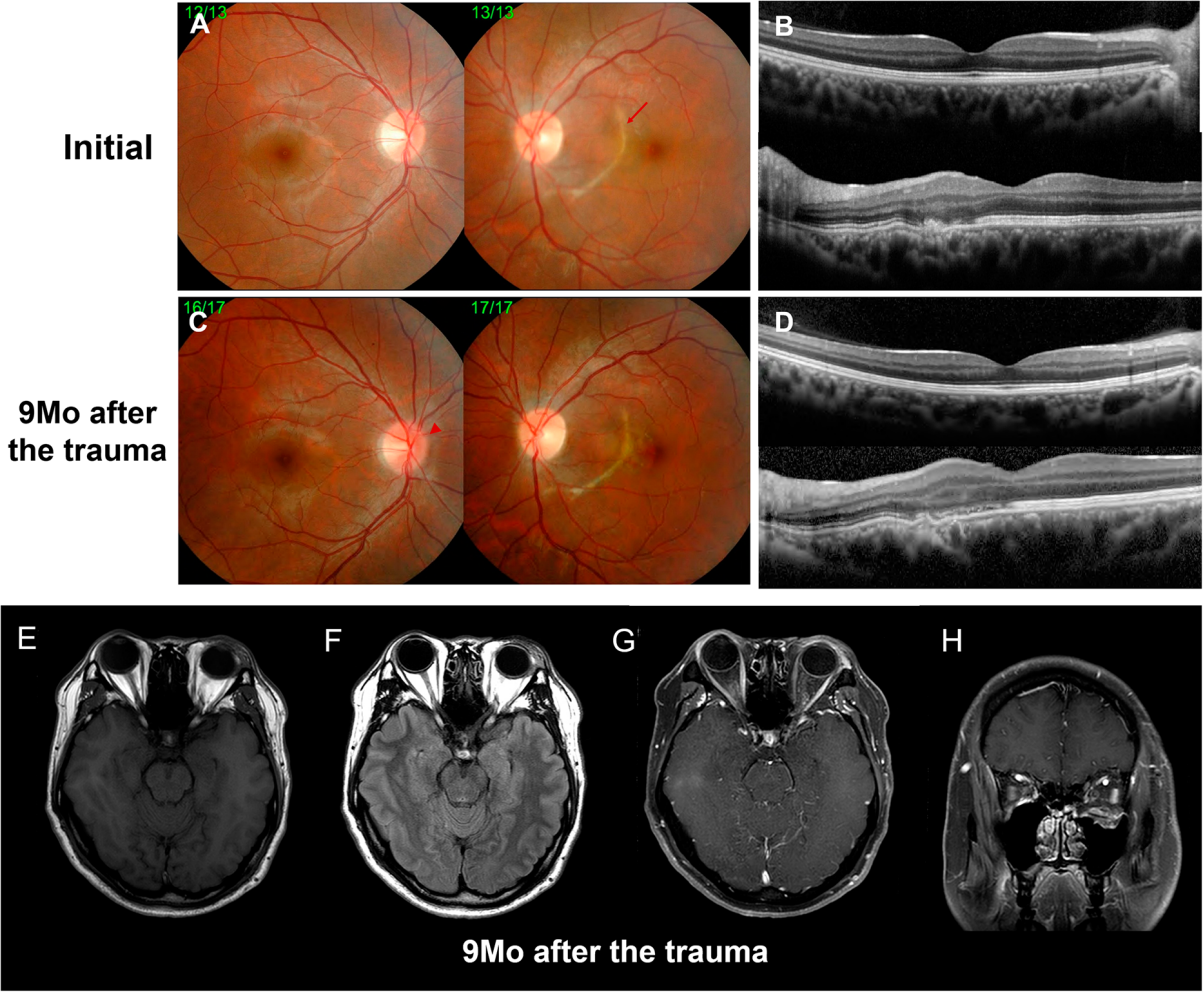
Comparison of fundus findings of the patient at initial visit and 9 months post-trauma, and a brain magnetic resonance imaging (MRI) with contrast enhancement that was performed after 9 months of the trauma. Initially, fundus photography (**a**, arrow) and optical coherence tomography (OCT) scan (**b**) revealed choroidal rupture in the left eye. However, the patient underwent central visual loss in the right eye 9 months post-trauma. No marked changes on the fundus (**c**) and OCT scan (**d**) are shown with the exception of the presence of a slightly hyperemic optic nerve head (arrowhead). No notable abnormal findings are shown in T1WI (**e**), T2WI (**f**), and contrast-enhanced MRI images (**e**–**h**)

Initially, his best-corrected visual acuity (BCVA) was 20/20 in the right eye and 20/1000 in the left eye. The intraocular pressures (IOPs) measured by Goldmann applanation tonometry were 16 mmHg in the right eye and 18 mmHg in the left eye. A relative afferent pupillary defect (RAPD) was apparent in the left eye, and traumatic hyphema was identified in the anterior chamber (Fig. 1d). A fundus examination revealed choroidal rupture that circumferentially crossed the macular area in the left eye (Fig. 1e), and temporal pallor was found in the left optic nerve head. Fundus fluorescein angiography (FFA) showed hyperfluorescence caused by a window defect, and indocyanine green angiography (ICGA) revealed hypofluorescence within the corresponding area of choroidal rupture (Fig. 1f, g). Horizontal optical coherence tomography (OCT) scan revealed choroidal rupture associated with interruption on retinal pigment epithelium layer, outer nuclear layer, and outer plexiform layer of the left eye (Fig. 2b), but the foveal area was relatively spared. BCVA did not improve despite high-dose steroid pulse therapy to treat traumatic optic neuropathy (TON) of the left eye, and temporal pallor of the optic nerve head was persisted. Thereafter, he underwent restoration surgery of the orbital wall, and regular follow-up was performed. Throughout the examination period, no specific findings were revealed on the fundus examination and OCT scan of the right eye (Fig. 2a, b).

**Fig. 3:**
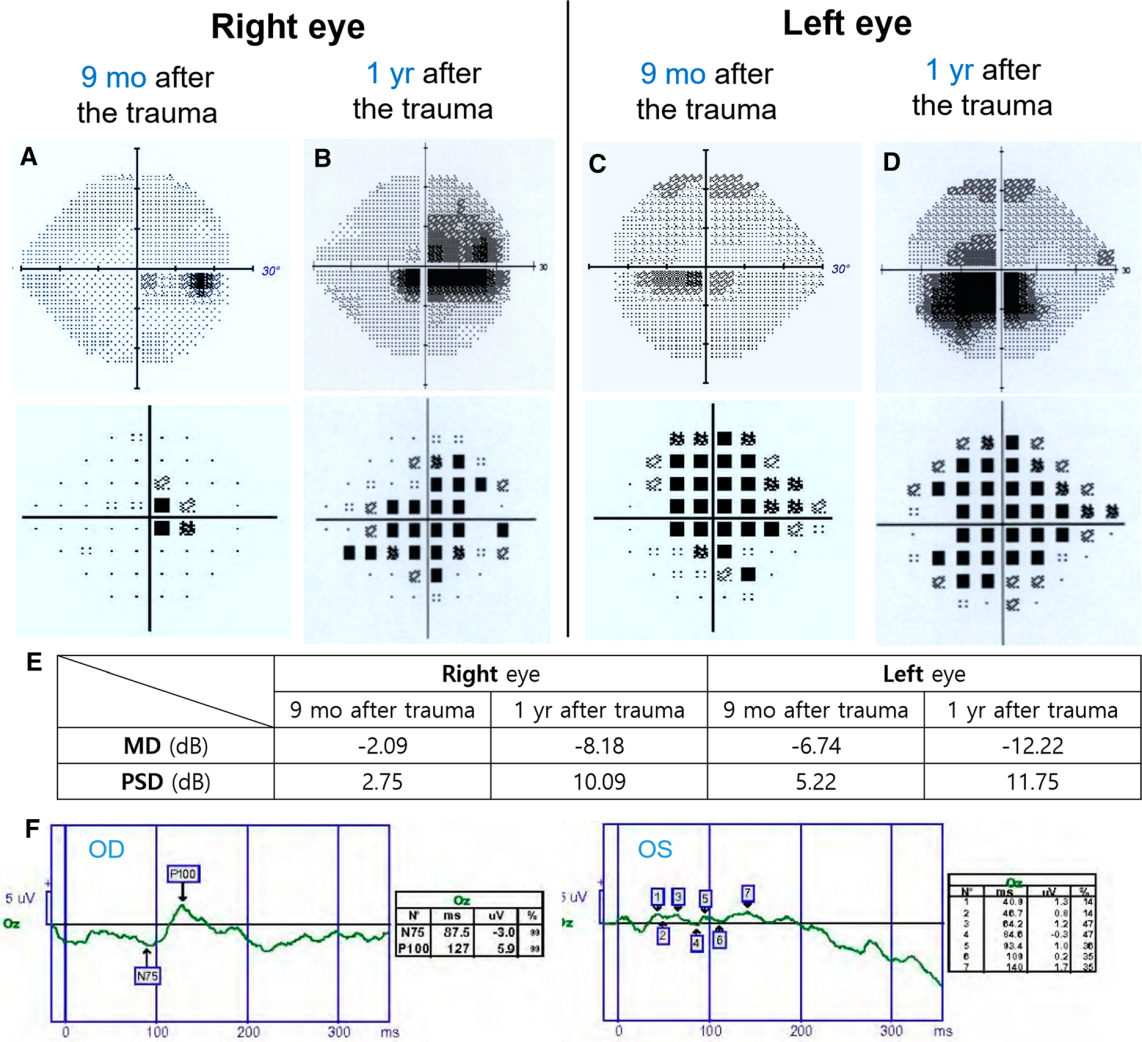
The changes in automated visual field (AVF) test findings and pattern visual evoked potential (PVEP) results that occurred in the patient between 9 and 12 months post-trauma. Central scotoma of the right eye worsened throughout the period considered (**a**, **b**), and the visual field defect of the left eye also enlarged after 12 months (**c**, **d**). Mean deviation (MD) and pattern standard deviation (PSD) of the AVF also deteriorated (**e**). Pattern VEP showed markedly decreased amplitudes and elongated latencies in both eyes between 9 months and 1 year post-trauma (**f**). The latency of P100 wave was 127 ms in the right eye, and that was indistinguishable from the noise level in the left eye

Nine months later, the patient complained with gradual central visual loss in the right eye, which was the contralateral to the ocular trauma. At that time, his BCVA was 20/200, and no remarked changes regarding fundus photography and OCT scan were observed with the exception of a slightly hyperemic optic nerve head of the right eye (Fig. 2c, d). In addition, automated visual perimetry (AVF) revealed cecocentral scotoma of the right eye (Fig. 3a, c). There were no notable abnormal findings observed via T1 weighted, T2 weighted, and contrast-enhanced images of brain magnetic resonance imaging (MRI) (Fig. 3e–h).

Twelve months after the accident, his BCVA in both eyes was reduced to 20/2000. Nine to 12 months after the injury, central scotoma of the right eye worsened, and the visual field defect of the left eye also enlarged after 12 months (Fig. 3b, d). Mean deviation (MD) of AVF decreased from − 2.09 to − 8.18 dB in the right eye and from − 6.74 to − 12.22 dB in the left eye. Additionally, pattern standard deviation (PSD) deteriorated from 2.75 to 10.09 dB in the right eye and from 5.22 to 11.75 dB in the left eye (Fig. 3e). Pattern visual evoked potential (PVEP) identified remarkably decreased amplitudes and elongated latencies in both eyes after 9 months to a year post-injury (Fig. 3f). The latency of P100 wave from pattern VEP was 127 ms in the right eye, and it was unmeasurable in the left eye because values were indistinguishable from noise level. Finally, mitochondrial DNA (mtDNA) analysis revealed a T14484C point mutation with a secondary mutation at C3497T. Therefore, the patient was diagnosed with LHON, and it was suggested that he experienced activation of LHON in unaffected LHON carrier followed by the trauma at fellow eye. No another specific treatment except vitamin B_12_ and C supplement administration was applied for the patient after being confirmed as LHON. The patient did not want additional treatment. Two years post-injury, his BCVA remained at 20/2000 in both eyes, and his central visual field defect did not recover.

## Discussion

Trauma-associated LHON or activation of LHON after trauma including skull fracture, intracranial hemorrhage, or blunt ocular trauma has been rarely reported previously [1, 2]. However, the specific mechanism in which the conversion of LHON occurs followed by trauma in unaffected carriers remains unclear. It has been proposed that traumatic insult may trigger acute decompensation of the optic nerve, which is already compromised by mitochondrial dysfunction [1]. It has also been suggested that post-traumatic intracranial superoxide levels can be associated with the conversion to symptomatic LHON via retrograde apoptosis of RGCs in the optic nerve [1, 2].

In this case, the patient reported no specific symptoms prior to the head injury and ocular trauma of fellow eye. After 9 months, he experienced visual loss with mild optic disk abnormality and displayed enlarged cecocentral scotoma. LHON was confirmed by the genetic analysis mitochondrial DNA mutation. Therefore, this case presents the healthy carrier of an LHON-associated point mutation in the mtDNA who experienced the activation of LHON post-ocular trauma of his fellow eye.

A previous report involved a case of trauma-associated LHON, which occurred after a kicking injury to the head. Optic disk swelling was revealed in the fundus examination, and enlargement of the optic chiasm was observed in the brain MRI [1]. The onset of visual loss by LHON after the direct trauma was the next day in the patient, and his BCVA reduced from 20/200 to count finger and did not recover [1]. Another report described activation of LHON in healthy carrier via trauma associated with being struck by a rope [2]. Although there were no abnormal findings in brain MRI, pallor with excavation of the optic nerve head was observed. Fortunately, the BCVA of the patient recovered from 8/200 to the normal range. The period from the trauma to onset of LHON in unaffected patient was 9 months, which was similar period with the current case [2].

Clinically, the case reported here displayed manifestation of LHON that was unique to those previously reported. In this case, visual loss and cecocentral scotoma were found after 9 months in the eye fellow to that which was injured by direct trauma. In addition, the scotoma of the eye directly injured by trauma was markedly enlarged after 12 months, which underwent a choroidal rupture by the trauma (Fig. 3).

Genetically, this patient also had unusual findings compared with other cases, and he had T14484C mutation. In both of the previous cases described above, on the other hand, results of mtDNA analysis revealed G11778A point mutations [1, 2]. It is widely known that the most common mtDNA mutation related to LHON is G11778A, while T14484C mutation revealed milder pathogenicity and is generally associated with better outcomes relative to other mutations [3, 4]. The patient described here had a T14484C point mutation with a secondary mutation of C3497T, which might produce a synergistic deleterious effect [5]. Clinical manifestations and genetic findings of this case were differed from other trauma-associated LHON patients. It is presumed that LHON was activated due to the trauma to the fellow eye and eventually resulted in visual loss and visual field defect progression in both eyes.

This report has some limitations. First, retinal nerve fiber layer (RNFL) loss is a crucial indicator of LHON. However, the measurement of RNFL thickness using OCT scan was not performed, because it had been thought that visual loss was induced by choroidal rupture on the macular area. Second, there is a possibility that the visual loss due to LHON already exists in the left eye. He had no familial history of LHON, and he did not undergo any other symptoms associated with LHON. At initial presentation, he revealed no specific sign on the optic nerve head, except mild temporal pallor. This report focused on the activation of LHON by trauma on the contralateral eye in unaffected patient, and visual loss by LHON was manifested on uninjured eye after several months. In addition, this report tried to present this unusual case with electrophysiological findings including PVEP.

In conclusion, the present case is unusual because the activation of LHON occurred in the eye that was contralateral to that which was injured, and head trauma also occurred on the opposite side. This observation suggests the possibility that the activation of LHON in healthy carriers may occur in patients who experience visual loss with optic neuropathy features after head or ocular trauma. Future research should be conducted to determine the precise mechanism that underlies the activation of unaffected LHON carriers after ocular or head trauma.
