# Correction to: Leber hereditary optic neuropathy following head trauma and ocular trauma on contralateral eye: a case report

**DOI:** 10.1007/s10633-020-09809-5

**Published:** 2021-01-21

**Authors:** Hoon Dong Kim

**Affiliations:** Department of Ophthalmology, College of Medicine, Soonchunhyang University, 30, Soonchunhyang 6-gil, Dongnam-gu, Cheonan-si, Chungcheongnam-do 31151 South Korea

## Correction to: Doc Ophthalmol 10.1007/s10633-020-09801-z

The original version of the article contained inaccuracies in the Ethics declarations.

The Ethical approval and the Statement of human rights are replaced by the following disclosure.

**Ethical approval**

All procedures performed in the case report involving human participant were in accordance with the ethical standards of Soonchunhyang University Cheonan Hospital and with Helsinki Declaration (1964) and its later amendments or comparable ethical standards. This study was approved by the Institutional Reviewer Board (IRB) of Soonchunhyang University Cheonan Hospital (IRB No. 2020-07-043).

Also the article reports on findings that were presented at the ISCEV Symposium in Miami in 2017 and published in our journal (1).
